# BIDIRECTIONAL ASSOCIATIONS BETWEEN PHYSICAL ACTIVITY AND AFFECT: AN ECOLOGICAL MOMENTARY ASSESSMENT STUDY

**DOI:** 10.1093/geroni/igae098.1444

**Published:** 2024-12-31

**Authors:** Peichao Liang

**Affiliations:** The University of Hong Kong, Pokfulam, Hong Kong

## Abstract

Despite extensive evidence demonstrating the beneficial effects of physical activity on health outcomes, many older adults remain physically inactive. A growing body of research examines affective states as both determinants and consequences of physical activity. However, the nuanced and dynamic relationships between physical activity and affect among older adults remain understudied. We used ecological momentary assessment (EMA) to investigate the bidirectional relationships between physical activity and affect in daily life among older adults in Hong Kong. 168 older adults aged 65 to 84 received seven EMA prompts per day for fifteen consecutive days and completed a total number of 17,345 momentary assessments of affective states, mobility, and activities. We used multilevel models to test the bidirectional associations between physical activity and positive affect, negative affect, and energetic arousal, nested within individuals. Preliminary results reveal concurrent associations between physical activity and positive affect (b=0.042, SE=0.008, p<.001) and energetic arousal (b=0.107, SE=0.027, p<.001). When older adults engaged in physical activity before the prompt, they reported a higher level of positive affect (b=0.038, SE=0.008, p<.001). However, physical activity did not significantly improve energetic arousal levels later (b=0.046, SE=0.027, p=0.089). Additionally, individuals were more likely to engage in physical activity after they reported a higher level of positive affect (OR=1.321, 95% CI=1.234-1.413, p<.001) and feeling energetic (OR=1.078, 95% CI=1.044-1.112, p<.001). No associations between physical activity and negative affect were identified. The implications of these findings for developing context-specific interventions to promote physical activity among older adults will be discussed.

